# Exome Sequencing Identifies *TENM4* as a Novel Candidate Gene for Schizophrenia in the SCZD2 Locus at 11q14-21

**DOI:** 10.3389/fgene.2018.00725

**Published:** 2019-01-28

**Authors:** Chao-Biao Xue, Zhou-Heng Xu, Jun Zhu, Yu Wu, Xi-Hang Zhuang, Qu-Liang Chen, Cai-Ru Wu, Jin-Tao Hu, Hou-Shi Zhou, Wei-Hang Xie, Xin Yi, Shan-Shan Yu, Zhi-Yu Peng, Huan-Ming Yang, Xiao-Hong Hong, Jian-Huan Chen

**Affiliations:** ^1^Mental Health Center, Shantou University Medical College, Shantou, China; ^2^Shantou Central Hospital, Affiliated Shantou Hospital of Sun Yat-sen University, Shantou, China; ^3^Laboratory of Genomic and Precision Medicine, Wuxi School of Medicine, Jiangnan University, Wuxi, China; ^4^Shenzhen Kang Ning Hospital, Shenzhen, China; ^5^Beijing Genomics Institute – Shenzhen, Shenzhen, China

**Keywords:** association, co-segregation, schizophrenia, exome analysis, rare mutation

## Abstract

Schizophrenia is a complex psychiatric disorder with high genetic heterogeneity, however, the contribution of rare mutations to the disease etiology remains to be further elucidated. We herein performed exome sequencing in a Han Chinese schizophrenia family and identified a missense mutation (c.6724C>T, p.R2242C) in the teneurin transmembrane protein 4 (*TENM4*) gene in the SCZD2 locus, a region previously linked to schizophrenia at 11q14-21. The mutation was confirmed to co-segregate with the schizophrenia phenotype in the family. Subsequent investigation of *TENM4* exons 31, 32, and 33 adjacent to the p.R2242C mutation revealed two additional missense mutations in 120 sporadic schizophrenic patients. Residues mutated in these mutations, which are predicted to be deleterious to protein function, were highly conserved among vertebrates. These rare mutations were not detected in 1000 Genomes, NHLBI Exome Sequencing Project databases, or our in-house 1136 non-schizophrenic control exomes. Analysis of RNA-Seq data showed that *TENM4* is expressed in the brain with high abundance and specificity. In line with the important role of *TENM4* in central nervous system development, our findings suggested that increased rare variants in *TENM4* could be associated with schizophrenia, and thus *TENM4* could be a novel candidate gene for schizophrenia in the SCZD2 locus.

## Introduction

Schizophrenia (OMIM 181500) is a complex psychiatric disorder, and is also a public health problem affecting approximately 1% of the world population, leading to reduced life expectancy by an average of 20–25 years (Tiihonen et al., 2009). Family and twin studies have demonstrated a strong genetic component in schizophrenia with heritability estimated to be 60–80% (Sullivan et al., 2003; Shih et al., 2004).

High heritability in the disease underlines substantial effects from genetic variants. The frequency of these of alleles ranges from common to extremely rare. Common variants associated with schizophrenia have been widely studied by candidate gene association studies (Stefansson et al., 2002; Chen et al., 2009a, 2) and genome-wide association studies (GWASs) (Bergen and Petryshen, 2012). For example, a recent multi-stage schizophrenia GWAS of up to 36,989 cases and 113,075 controls identify 128 independent associations spanning 108 loci that meet genome-wide significance (Schizophrenia Working Group of the Psychiatric Genomics Consortium, 2014). It is estimated that half to a third of the genetic risk of schizophrenia is indexed by common alleles in GWAS.

Apart from common variants with small effect, recent studies have shown that rare variants or mutations with large effect may also help to understand remaining component of psychiatric etiology unexplained by common variants (Pulver et al., 1994;Jacquet et al., 2002; Ament et al., 2015). For example, a mutation in the Neuronal PAS domain protein 3 (NPAS3) gene segregates with mental illness in a family affected by schizophrenia and major depression (Yu et al., 2014, 3). Recent exome sequencing studies have advanced understanding of rare variants and mutations in schizophrenia (Bustamante et al., 2017; Singh et al., 2017; John et al., 2019). Studies in parent-proband trios have revealed *de novo* mutations with high genetic heterogeneity in schizophrenia (Xu et al., 2011, 2012). Moreover, analysis of exomes from 2536 schizophrenia cases and 2543 controls has emphasized burden raised from extremely rare (less than 1 in 10,000), disruptive mutations in the patients (Purcell et al., 2014). However, the contribution of rare mutations to schizophrenia remains to be further elucidated. In spite of a number of genetic loci identified by linkage studies in schizophrenia,^1^ only a few genes have been mapped in these loci, such as *PRODH* (SCZD4) (Jacquet et al., 2002), *DISC1* (SCZD9) (Debono et al., 2012, 1), *SHANK3* (SCZD15) (Gauthier et al., 2010), *NRXN1* (SCZD17) (Rujescu et al., 2009), and *SLC1A1* (SCZD18) (Myles-Worsley et al., 2013, 1). Most of these linkage loci have not yet been defined molecularly, which might harbor unknown genes or mutations with large effects in disease risk determination that remain to be identified.

In the current study, by using exome sequencing we identified a novel rare mutation in the teneurin transmembrane protein 4 gene (*TENM4*, also named *ODZ4*), which cosegregated with schizophrenia in a Han Chinese family. Moreover, additional rare *TENM4* mutations were found in a cohort of 120 unrelated sporadic schizophrenic patients. All mutation was not detected in 1441 non-schizophrenic control exomes. *TENM4* is located within the schizophrenia disorder 2 (SCZD2) locus at 11q14-21, in which the underlying gene has not been identified by far. The study thus demonstrated increased *TENM4* mutation burden in schizophrenia and suggested that *TENM4* could probably be a candidate gene for schizophrenia in the SCZD2 locus.

## Materials and Methods

### Sample Collection and Clinical Examination

**FIGURE 1:**
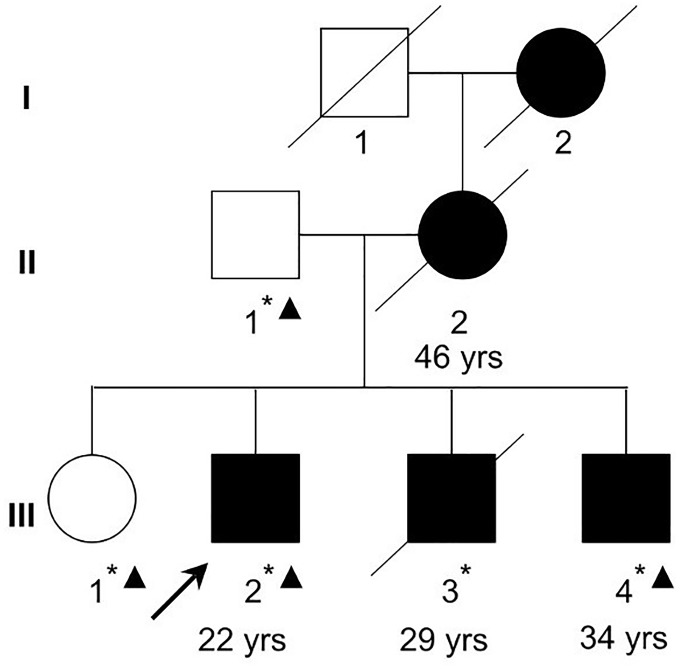
A Chinese Han family with schizophrenia. Filled squares and circles denote affected males and females, respectively. Normal individual is shown as empty symbols. All family members in the second and third generations were examined. The ages at diagnosis for the examined affected family members are shown below the symbols. Whole-exome sequencing was performed in two affected (III-2 and III-4) and two unaffected (II-1 and III-1) family members. Asterisks denote individuals with blood samples and DNA collected. Triangles denote individuals (II-1, III-1, III-2, and III-4) whose DNA were used in exome sequencing in the current study.

This study has been approved by the Ethics Committee of the Mental Health Center of Shantou University Medical College and was performed in accordance with the ethical standards laid down in the 1964 Declaration of Helsinki and all subsequent revisions. All participants in the paper gave their informed consent for their participation, and the publication of clinical data and indirectly identifiable information prior to their inclusion in the study. The family with schizophrenia was recruited at the Mental Health Center of Medical College of Shantou University, Shantou, China (Figure 1). Clinical diagnoses in the proband (III-2) and family members were based on a Chinese version of the Structured Clinical Interview for DSM-IV-TR Axis I Disorder-Patient Edition (SCID-I/P) (First et al., 1997) criteria derived from a standard interview and from a case-note review by two trained psychiatrists. Four affected family members (II-2, III-2, III-3, and III-4) were interviewed with Social Disability Screening Schedule (SDSS) (World Health Organization, 1988) to assess the clinical-specific features and social abilities. Clinical global impression-severity of illness (CGI-SI) (Programs and Guy et al., 1976) was used to assess the disease severity (Table 1). All of them were diagnosed as paranoid schizophrenia and had social or occupational dysfunction and continuous signs of the disturbance for more than 15 years. Four individuals affected with treatment-resistant schizophrenia were enrolled. The two unaffected family members were confirmed to have no sign of mental illness.

**Table 1:** Demographic information and clinical features of four affected family members from schizophrenia family enrolled in the exome sequencing study.

Item	I-2	II-2	III-2	III-3	III-4
Sex	F	F	M	M	M
Age at diagnosis (years)	NA	46	22	29	34
Age at last interview (years)	NA	71	47	44	49
Delusions	NA	Yes, jealousy	Yes, persecution	Yes, persecution and grandeur	Yes, persecution and grandeur
Hallucination	NA	Yes, auditory	Yes, auditory	Yes, auditory	Yes, auditory
Disorganized thinking (Speech)	NA	Yes, mild	Yes, prominent	Yes, prominent	Yes, prominent
Abnormal motor behavior (Catatonia)	NA	Yes, mild	Yes, prominent	Yes, prominent	Yes, prominent
Negative symptoms	NA	Yes, prominent	Yes, prominent	Yes, severe	Yes, prominent
Substance use	NA	No	No	No	No
SDSS score	NA	14	12	11	13
CGI-SI score	NA	6	5	4	6
Course specify	NA	Continuous	Continuous	Continuous	Continuous
Latest medical condition	NA	Deceased (cardiovascular disease)	Deceased (liver cancer)	Deceased (sudden death)	Hospitalized
Age of death (year)	NA	75 (2008)	57 (2011)	50 (2010)	NA

*M, male; F, female; NA, not available or applicable; SDSS, Social Disability Screening Schedule; CGI-SI, clinical global impression-severity of illness.*

A cohort of 120 unrelated sporadic schizophrenic patients was recruited using the same criteria as described above. All of them were diagnosed as schizophrenia, and their main manifestations included auditory hallucination, persecutory delusion, and social or occupational dysfunction.

A group of 205 unrelated non-schizophrenia individuals in Sanger sequencing validation had no history of psychotic diseases following clinical interview FH-RDC criteria (Endicott, 1978).

Peripheral blood was collected from all participants except for II-2 whose blood and DNA was not available due to her death before blood sample collection for the current study. Her genotypes and mutation status was then inferred from her children and spouse’s genotypes. Genomic DNA was extracted by using the QIAmp Blood kit (Qiagen, Hilden, Germany).

### Exome Capture and Sequencing

Genomic DNA (3 μg) from two affected (III-2 and III-4) and two unaffected members (II-1 and III-1) was used to perform exome sequencing by Axeq Technologies (Rockville, MD, United States). Whole exome was captured by SeqCap EZ Human Exome Library v3.0 (Roche NimbleGen, Madison, WI, United States) and sequenced on an Illumina HiSeq 2000 (Illumina, Hayward, CA, United States) with a paired-end 100 bp length configuration.

### Read Mapping and Variant Detection

The reads were mapped against UCSC hg19 Human Reference Genome^2^ by using BWA^3^. The single nucleotide variations (SNVs) and Indels were detected by SAMTOOLS^4^, and annotated using ANNOVAR (Wang et al., 2010) previously known and reported variants were identified and filtered using dbSNP 135^5^ and 1000 Genomes project^6^ data. Functional impact of variants was predicted by PROVEAN, SIFT, and PolyPhen.

### Sanger Sequencing

Genomic sequence of *TENM4* was obtained from the NCBI reference sequence database^7^. Primers designed accordingly by Primer 3 were summarized in Supplementary Table S5. Polymerase chain reaction (PCR) amplification was performed using the GeneAmp PCR System 9700 (ABI, Foster City, CA, United States) in a 25-μl mixture containing 1.5 mM MgCl_2_, 0.2 mM of each dNTP (Sangon, Shanghai, China), 1 U Taq DNA polymerase (Invitrogen, Carlsbad, CA, United States), 0.2 μM primers, and 20 ng of genomic DNA. Sanger sequencing was performed using the BigDye Terminator Cycle Sequencing v3.1 kit (ABI, Foster City, CA, United States) and the 3130xl Genetic Analyzer (ABI, Foster City, CA, United States) following the protocol suggested by the manufacturer. Sequence alignment and analysis of variations were performed using the NovoSNP program^8^.

### Non-schizophrenic Control Exomes

Variants were screened against exomes from Chinese Han individuals without schizophrenia to remove common variants. The set of control exome data were consolidated from several previous exome sequencing studies performed by our group. Various exome capture chips were used for this dataset, such as Roche NimbleGen SeqCap EZ Human Exome Library v3.0, Agilent SureSelect All Human Exon v4.0 kit, or Ilumina TruSeq Exome Enrichment kit. Sequencing was performed in Illumina Hiseq 2000. The data quality criteria and filtering process followed the same standard as that for the family.

### Statistical Analysis

Difference in scores of clinical assessment was analyzed using Student’s *t*-test. Association of SNVs with schizophrenia was performed using Fisher’s exact test.

### RNA-Seq of Human Tissues

Fragments Per Kilobase of transcript per Million mapped reads (FPKM) values of RNA-seq of the brain, cerebellum, heart, kidney, liver, and testis from human, *macaque*, mouse, and opossum were obtained from the Baseline Atlas of Gene Expression Altas website^8^ (Keane et al., 2011). Relative expression level of *TENM4* mRNA was calculated as the original expression normalized by the highest expression among the six tissues in each species.

## Results

### Clinical Data of the Schizophrenia Family

A Chinese Han family of five family members with three generations affected by schizophrenia was recruited from our longitudinal follow-up, and latest interview was done in 2004 for II-2 and III-3, and 2011 for III-2 and III-4, respectively (Figure 1, Table 1). All affected family members received the interviews were all diagnosed as schizophrenia (Table 1). The affected patients all had disease-onset after adulthood (age of onset later than 22 years old), and manifested auditory hallucination, persecutory delusion, and were easy to be offended or irritated and aggressive in the acute stage. They developed gradually into difficulty in concentration, and blunting affect, leading to social isolation and work disability for multiple episodes.

### Exome Sequencing Data of the Family

In order to identify the underlying genetic predisposition in the family, exome sequencing was performed in two affected (III-2 and III-4) and two unaffected (II-1 and III-1) family members. The original exome sequencing data from the four family members were summarized in Supplementary Table S1. The exome sequencing achieved 96.2% mean coverage and 62.5× mean depth of target region, which allowed high quality of variant calling (Supplementary Table S2). The variants called from the exome data were analyzed using a step-by-step filtering method (Table 2). The variants were firstly filtered to remove all noncoding and synonymous variants, and keep only nonsynonymous single-nucleotide variants (NSVs), splice site variant (SSV), and coding indels. The selected variants were then filtered against databases including dbSNP 135, 1000 Genomes, the NHLBI Exome Sequencing Project, and non-psychiatric exomes from the Exome Aggregation Consortium (ExAC) to remove common and known variants in the public databases. Given the pattern of dominant inheritance in the family, only heterozygous variants shared by the two affected (III-2 and III-4) but not found in the two unaffected (II-1 and III-1) were kept for subsequent analysis. In order to look for rare variants, the remaining variants were then filtered against exomes of non-schizophrenic controls. Twenty-eight remaining variants had a MAF less than 1% in the control exomes (Table 2), none of which was in *PRODH*, *DISC1*, *SHANK3*, *NRXN1*, or *SLC1A1*. These variants were then evaluated for impact on protein function to by three bioinformatic programs PROVEAN, SIFT, and PolyPhen-2. Higher priority was given to four NSVs and one SSV that were predicted to be deleterious and were not detected in control exomes (Table 2). These five variants were then analyzed with prior knowledge of gene function as shown in Supplementary Table S4. *SLC11A2* is a known disease-causing gene for autosomal recessive anemia (OMIM #206100) (Iolascon et al., 2006). Likewise *TSPAN12* has been characterized as a disease-causing gene for exudative vitreoretinopathy (OMIM #613310) (Nikopoulos et al., 2010). These two genes were known disease-causing genes of distinct phenotypes from schizophrenia. A recently study on autozygosity showed lack of an apparent phenotype in individuals with loss of function variants of FUK (Alsalem et al., 2013). Therefore, these three genes were excluded from further study. *TENM4* is located within a region that was previously linked to schizophrenia (SCZD2, OMIM %603342). *LRRTM4* has highly selective expression in the brain and can mediate excitatory synapse development on dentate gyrus granule cells (Siddiqui et al., 2013). Therefore, *TENM4* and *LRRTM4* were then included for subsequent validation experiment.

**Table 2:** Prioritized deleterious rare mutations identified by exome sequencing.

Filters	III-2	III-4	III-2 + III-4	Heterozygous and with MAF ≤ 1% in control exomes	Heterozygous and not in control exomes	PROVEAN^*a*^	SIFT^*b*^	Polyphen-2^*b*^	PROVEAN + SIFT + PolyPhen-2	SSV	Within linkage regions
NSVs, SSVs, and coding indels	9745	9955	5958	522	196						
Not in dbSNP 135	616	638	209	169	109						
Not in dbSNP 135 or 1000 Genomes	537	561	172	148	103						
Not in dbSNP 135, 1000 Genomes, NHLBI ESP	522	543	162	140	98						
Not in dbSNP 135, 1000 Genomes, NHLBI ESP, or unaffected (II-1 or III-1)	242	282	36	28	14	6	8	9	*4 (TENM4, FUK, LRRTM4, TSPAN12)*	1 (*SLC11A2*)	1 *(TENM4*)*^c^*

*NSVs, nonsynonymous single-nucleotide variants; SSVs, splice site variants; ^a^NSVs or indels predicted by PROVEAN to be deleterious. ^b^NSVs predicted by PROVEAN, SIFT, and PolyPhen-2 to be deleterious or damaging. ^c^Among the four genes, only TENM4 was located within a region at 11q14-q21 linked to schizophrenia.*

### Cosegregation With Schizophrenia in the Family

**FIGURE 2:**
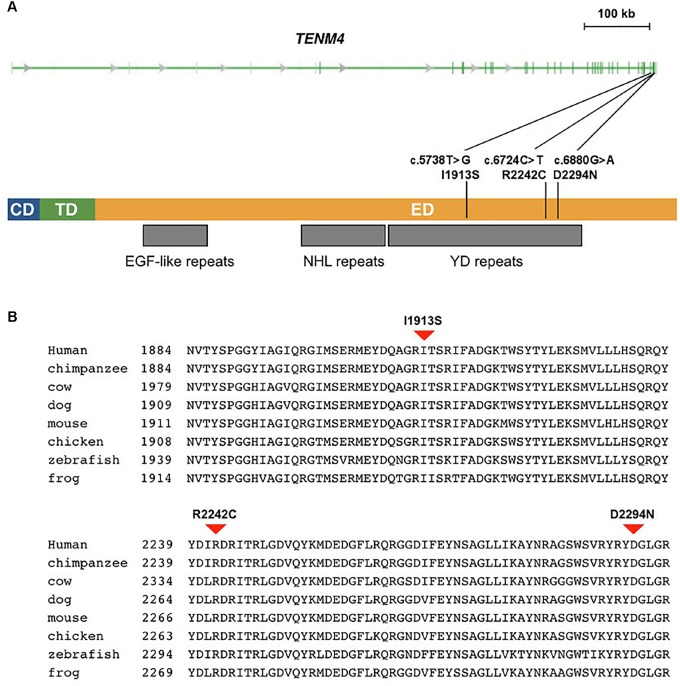
Missense mutations identified in schizophrenia patients in *TENM4* at 11q14.1. **(A)** The genomic features of the *TENM4* gene and the domains in the encoded protein. TENM4 contains a cellular domain (CD), a transmembrane domain (TD), and a large extracellular domain (ED) containing EGF-like, NHL repeats, and YD repeats (indicated underneath the protein). The three mutations detected in schizophrenic patients are shown. **(B)** Sequence alignment of TENM4 orthologs in vertebrates. The residues (I1913, R2242, and D2294) mutated in the three mutations were conserved among vertebrates from zebrafish through human. The three mutations were predicted to be deleterious to the protein function.

Sanger sequencing results showed that the *LRRTM4* variant was not found in one affected family member III-3, and thus were excluded from further analysis due to lack of co-segregation with the disease phenotype. The c.6724C>T variant in exon 32 of *TENM4* was heterozygous in the four affected (III-2, III-3, and III-4 by sequencing and II-2 by inferring) (Figure 2A and Supplementary Figure S1), and were not detected in the two unaffected (II-1 and III-1). These results showed that the c.6724C>T variant cosegregated with the schizophrenia phenotype in the schizophrenia family. The variant was not detected in any of another group of 205 unrelated non-schizophrenic controls by Sanger sequencing, in consistent to its absence in the public databases and our in-house control exomes as described above.

### *TENM4* Mutations in Unrelated Sporadic Schizophrenic Patients

As *TENM4* could possible serve as mutation hotspots, we further investigate *TENM4* exons 31, 32, and 33 which were adjacent to c.6724C>T in 120 unrelated sporadic schizophrenic patients using Sanger sequencing (Supplementary Table S5). Two additional missense alterations, c.5738T>G (p.I1913S) in exon 31 and c.6880G>A (p.D2294N) in exon 32 were detected (Table 3), both of which were predicted to be deleterious to protein function by PROVEAN, SIFT, and PolyPhen-2. The variant p.D2294N was found in a single heterozygous individual (MAF = 8.3 × 10^−6^) in the ExAC database but not found in our controls. And p.I1913S was not detected in the public databases, control exomes, or in the Sanger sequencing results of 205 unrelated controls. A rare synonymous variant (p.K1922K) was also significantly associated with schizophrenia (*p* < 0.05). In contrast, only one missense variant with a MAF less than 0.04% (1 in 2882) in the YD repeats was detected in the same exons in controls, which was predicted to be benign, and was not significantly different between patients and controls (*p* = 1). Further comparison of clinical features did not find evident difference between patients with and without either of p.I1913S and p.D2294N.

**Table 3:** Additional variants in unrelated schizophrenic patients and controls in *TENM4* exons adjacent to the mutation found in the family.

No.	Exon	cDNA change	Amino acid change	Consequence	dbSNP 135 ID	SIFT prediction	PolyPhen prediction	Genotype (MM/Mm/mm)			Allelic association
								SCZ	Con (Sanger)	CON (exome)	*p*-value^∗^
1.	31	c.5535C>T	p.R1845R	Synonymous	rs111604043	NA	NA	120/0/0	205/0/0	1133/3/0	NC
2.	31	c.5738T>G	p.I1913S	Missense	Novel	Deleterious	Damaging	119/1/0	205/0/0	1136/0/0	NC
3.	31	c.5766G>A	p.K1922K	Synonymous	Novel	NA	NA	117/3/0	202/3/0	1136/0/0	<0.01
4.	32	c.6105C>T	p.D2035D	Synonymous	rs61740650	NA	NA	120/0/0	NA	1135/1/0	NC
5.	32	c.6114A>G	p.A2038A	Synonymous	rs61742000	NA	NA	118/2/0	NA	1129/6/1	0.247
6.	32	c.6627C>A	p.D2209E	Missense	Novel	Tolerated	Benign	120/0/0	205/0/0	1135/1/0	NC
7.	32	c.6651C>T	p.Y2217Y	Synonymous	rs61747204	NA	NA	120/0/0	205/0/0	1129/6/1	0.247
8.	32	c.6880G>A	p.D2294N	Missense	Novel	Deleterious	Damaging	119/1/0	205/0/0	1136/0/0	NC
9.	32	c.7062T>C	p.D2354D	Synonymous	rs17136977	NA	NA	120/0/0	NA	1133/3/0	NC
10.	32	c.7185C>T	p.N2395N	Synonymous	Novel	NA	NA	119/1/0	NA	1136/0/0	NC
11.	32	c.7191G>A	p.Q2397Q	Synonymous	rs61745709	NA	NA	120/0/0	NA	1135/1/0	NC

*SCZ, unrelated schizophrenic patients; CON, non-schizophrenic controls; MM, homozygous wild type; Mm, heterozygous mutation; mm, homozygous mutation; NA, not available; NC, not calculated. ^∗^Allelic association was done using Fisher’s exact test.*

### *TENM4* Residues Mutated in Schizophrenia

The human *TENM4* protein sequence was aligned to its orthologs in other vertebrates. The alignment showed that the *TENM4* residues mutated in schizophrenic patients were evolutionarily conserved (Figure 2). They were all located within a region that consisted of 23 tyrosine/aspartic acid (YD) repeats in the extracellular domain of the transmembrane *TENM4* protein.

To evaluate burden of rare variants in the gene, all coding variants of *TENM4* were examined in the exomes of the family and non-schizophrenia controls. The data were summarized in Supplementary Table S6. In total, 39 variants were detected in the gene from the exomes including the novel mutation pR2242C exclusively found in the affected family members. We analyzed the distribution of variants predicted to be deleterious in control exomes. Three novel rare variants that were predicted to damaging were exclusively found in control exomes, including a missense variant (p.R325W) in the teneurin N-terminus, a missense variant (p.E1098G) between the EGF-like and NHL repeats, and one stop-gained variant (p.Q2735X) in the last exon close to the C-terminus. However, by carefully looking into the positions of novel and known variants predicted to be deleterious in control exomes, none of them was found to be within the YD repeats. The three control individuals carrying these variants had no symptom of schizophrenia. Moreover, either control exomes or the two unaffected did not have any variant predicted to be deleterious in the YD repeats. In contrast, novel, rare deleterious variants in the YD repeat region were exclusively observed in affected family members and unrelated patients. These findings might suggest increased burden of rare deleterious variants within the YD repeats of *TENM4* was associated with schizophrenia (variant burden test *p* < 0.001).

### Expression Specificity of *TENM4* in the Brain

We then analyzed RNA-seq data of six human tissues including brain, cerebrum, heart, liver, kidney, and testis, which were retrieved from Gene Expression Atlas. The results showed that *TENM4* was highly expressed in human brain tissues (Supplementary Figure S2). In addition, the gene exhibited higher specificity to the brain compared to other tissues. Moreover, highest expression level of *TENM4* in the brain among six different tissues was observed in mouse, opossum, and monkey, suggesting that the high specificity of *TENM4* expression in the brain was evolutionarily conserved in mammals.

## Discussion

In the current study, we conducted exome sequencing in a three-generation family affected by schizophrenia, and identified a novel candidate gene *TENM4* in a linkage locus SCZD2 at 11q14-21. One missense mutation cosegregated with the schizophrenia phenotype, and two more missense mutations were detected in unrelated sporadic schizophrenic patients. These findings hence implicated a potential role of *TENM4* in etiology of schizophrenia.

The SCZD2 locus at 11q14-21 has not yet been defined molecularly for any disease gene for schizophrenia (Yamada et al., 2004). A balanced t(1;11)(q42.1;q14.3) translocation was first reported to co-segregate with schizophrenia (Millar et al., 2000). The 11q14-21 region has been linked to mental illnesses, including schizophrenia and bipolar disorder (Fanous et al., 2012). A recent study has further demonstrated that polymorphisms in the SCZD2 locus are associated with schizophrenia in Scottish population (Debono et al., 2012), emphasizing the possibility that the SCZD2 region may harbor genetic variants contributing to risk of schizophrenia. By showing co-segregation with schizophrenia in the family, our findings thus suggested that *TENM4* could be a candidate gene in the SCZD2 locus.

*TENM4* is located at 11q14.1 and contains 34 exons, which encode a large protein composed of 2769-amino acid residues. The molecular function of the gene has not yet been well characterized by far. It probably plays as a type II transmembrane protein, and functions as a cellular signal transducer. *TENM4* is a member of the TENEURIN/ODD OZ (TEN/ODZ) protein family, which is first identified in Drosophila (Baumgartner and Chiquet-Ehrismann, 1993; Baumgartner et al., 1994), and contains many members with orthologs identified in mammals, vertebrates, insects, and nematodes (Minet and Chiquet-Ehrismann, 2000). This family of transmembrane proteins shares a common TENEURIN N-terminus intracellular domain, 5–8 EGF-like repeats, and more than 20 tyrosine/aspartic acid (YD) repeats. *TENM4* were highly expressed in mammalian brain (Zhou et al., 2003). *TENM4* mutations in mice can be lethal, and the mutants exhibit anomalies in gastrulation (Lossie et al., 2005). It is noted that missense mutations in *TENM4* have recently been reported to cause autosomal-dominant essential tremor (Hor et al., 2015). *TENM4* rare variants are also found to be associated with bipolar disorder (Ament et al., 2015). Furthermore, a role of *Tenm4* has been implicated in a rodent model of schizophrenia (Neary et al., 2017). These studies taken together were in line with a substantial role of *TENM4* in mammalian central nervous system development and functions.

In the current study, a rare missense mutation (c.6724C>T; p.R2242C) in *TENM4* showed cosegregation with schizophrenia in a three-generation family. In addition, two more rare mutations were identified in unrelated sporadic schizophrenic patients (Table 3), suggesting the exon surrounding p.R2242C in *TENM4* could be a potential mutation hotspot for schizophrenia. Such findings thus pointed to potential association of *TENM4* with schizophrenia. It was noted that more *TENM4* missense were observed in these schizophrenic patients than in controls. It could be due to higher clinical homogeneity in our patients. All of the unrelated schizophrenic patients in the current study were diagnosed as paranoid schizophrenia. It should also be noted that the local population in the current study could be more genetically homogeneous as reported in a recent study (Chen et al., 2009b). The absence of rare *TENM4* mutations in non-schizophrenic controls was confirmed by using Sanger sequencing in 205 controls plus exome sequencing in a larger control cohort, which should ensure that the extreme rareness of these mutations was unlikely due to technical bias if any. Among the three *TENM4* mutations found in schizophrenia patients, p.D2294N was found in a heterozygous individual (MAF = 8.3 × 10^−6^) in the ExAC database indicated that these deleterious *TENM4* mutations might exist in the general population, yet with an extremely low frequency. We further checked loss-of-function variants in *TENM4* in the ExAC database. In total six loss-of-function variants were found for the canonical *TENM4* isoform, each of which was detected in a single heterozygous individual (MAF = 8.3 × 10^−6^), suggesting that loss-of-function variants in *TENM4* were extremely rare. For comparison, there were 27 loss-of-function variants found in *DISC1* in the ExAC database, with the highest MAF = 0.0005. Therefore, the function of TENM4 protein could be relatively conserved and was unlikely to be tolerant to loss-of-function mutations.

The TENM4 protein contains 1 teneurin N-terminal domain, 8 EGF-like repeats, 5 NHL (NCL-1, HT2A, and Lin-41) repeats, and 23 YD repeats. Our findings suggested that distribution of variants predicted to be deleterious was unlikely random. The mutations detected in schizophrenia patients in the current study were located within a YD repeat containing region in the extracellular domain of *TENM4* protein, which was essential for extracellular protein–protein interaction and signal transduction for transmembrane proteins. In contrast, only a benign missense variant with low MAF, and no deleterious variant were found in the YD repeat region among non-schizophrenia control exomes. A premature termination codon variant was identified in the last exon close to the C-terminus in one of our control exomes. However, it was not within the YD repeat. Encoding 98.7% of full-length protein, the variant was unlikely to trigger nonsense-mediated decay of mRNA to affect the protein function (Cirulli et al., 2011). Such findings suggested that increased burden of rare deleterious variants in YD repeats of *TENM4* was probably associated with schizophrenia. The YD repeat sequences contain two tandem copies of a 21-residue extracellular repeat named for a YD dipeptide, the most strongly conserved motif of the repeat (Minet et al., 1999). These repeats appear in general to be involved in binding carbohydrate, and their function has not been understood clearly. In human, YD repeats are found in all four of TENM proteins. YD repeats are found in all four of TENM proteins. Prior studies suggest that these conserved repeats might be involved in neuron development. Minet et al. (1999) reported that culture substrate coated with the YD repeat region of chicken teneurin-1 supported neuron outgrowth from dorsal root ganglion explants. Therefore, defects in YD repeats might contribute to development of psychiatric diseases due to its role in neuron development. And our results thus warranted further studies of the YD repeats and *TENM4* in molecular mechanism of schizophrenia.

Although *TENM4* has not been clinically linked to schizophrenia previously, recent studies have already implicated its possible role in mental illness and cognition. In an epigenetic study, the *Tenm4* gene has recently been implicated in a rodent model of schizophrenia (Neary et al., 2017). In addition, *TENM4* is associated with bipolar disorder in a recent genome-wide association study with a large sample size (Sklar et al., 2011). And genetic studies have shown prominent genetic overlap between schizophrenia and bipolar disorder. In a large population-based study in Sweden and Demark (Lichtenstein et al., 2009), risk of bipolar disorder was associated with a family history of schizophrenia. Moreover, a recent study has reported that rare variants in *TENM4* might contribute to etiology of bipolar disorder (Ament et al., 2015). Moreover, in a study on genetic correlation on brain magnetic resonance imaging and cognitive tests, *TENM4* has been ranked among the top 25 loci with Boston Naming test score via generalized estimating equations, suggesting its link to cognition function^33^. Association of cognitive impairment with schizophrenia has been implicated in previous studies (O’Carroll, 2000). Although our study subjects have not been assessed for cognition function directly in our sample collection, our records showed that all affected in the family received education and had been employed, suggesting they were unlikely to have cognitive impairment before disease onset. After disease onset, as the disease progressed, they gradually exhibited cognitive impairments with marked negative symptoms including disorganized thinking, lack of judgment and insight, social dysfunction, and loss of ability to work. Likewise, all of the unrelated schizophrenia patients had evident hallucination and delusion during the acute phase. As the disease progressed, cognitive impairments were observed in these patients including poor attention, active social avoidance, lack of judgment and insight, poor rapport, and disturbance of volition. The rare deleterious *TENM4* variants found in schizophrenia patients might not affect cognition directly. Therefore, our data suggested association of *TENM4* with schizophrenia, while its association with cognitive impairment needs to be investigated in further study. Nevertheless, taken together with these previous studies, our findings were in line with a substantial role of *TENM4* in central nervous system development and diseases.

Schizophrenia is of high genetic heterogeneity, and genetic variants with different effects can contribute to the disease risk. In addition to common variants with relatively small effects identified by both GWAS and candidate gene studies, the role of rare variants or mutations with large effects has been emphasized in psychiatric disorders such as autism (Chaste et al., 2014), especially by exome sequencing studies (Cukier et al., 2014; Toma et al., 2014). Schizophrenia-associated rare mutations have been depicted in recent exome sequencing studies (Purcell et al., 2014). However, most of these variants need to be further confirmed, and for most of them validation still remain a big challenge due to their low frequency (<1%) and uncertain penetrance, especially for schizophrenia. The mapping of rare mutations can be enhanced by analyzing rare variants cosegregated with disease phenotypes in high-density families of schizophrenia, and by integrating prior knowledge of linkage loci. The association between TENM4 mutations with schizophrenia in the current study was not contradictory to previous literature regarding architecture of schizophrenia genetic risk. First, to the best of our knowledge, mutations with large risk to schizophrenia have been reported in at least five genes, *DISC1*, *NRXN1*, *PRODH*, *SHANK3*, and *SLC1A1* according to records in OMIM (Supplementary Table S3). Second, the observation of *TENM4* mutations in some of our schizophrenic patients does not rule out possible contribution of other genetic factors such as polymorphisms with small effects or mutations with large effects to the disease in the same patients.

In the current study, by using exome sequencing, we demonstrated cosegregation of a novel missense mutation in *TENM4* with schizophrenia in a Chinese family. With additional rare *TENM4* mutations significantly associated with schizophrenia, our findings suggested that *TENM4* were a candidate gene for the disease in the SCZD2 locus at 11q14-21. Actual specific functional effects of these *TENM4* rare variants were thus warranted in further study.

## Additional Information

### Accession Codes

Whole-exome sequence data for the schizophrenia family has been deposited in GenBank Sequence Read Archive (SRA) under the accession code SPR172436.

### URLs

Online Mendelian Inheritance in Man (OMIM), http://omim.org/; PROVEAN, http://provean.jcvi.org/; SIFT, http://sift.jcvi.org/; PolyPhen-2, http://genetics.bwh.harvard.edu/pph2/; Gene Expression Atlas, http://www.ebi.ac.uk/gxa/; ExAC, http://exac.broadinstitute.org/.

## Author Contributions

C-BX and X-HZ contributed equally to this work. X-HH and J-HC conceived the project and planned the experiments. C-BX, X-HZ, YW, Q-LC, C-RW, J-TH, H-SZ, and W-HX clinically characterized the cases and collected blood samples. C-BX, Z-HX, and JZ performed the validation experiments. J-HC, XY, S-SY, Z-YP, and H-MY analyzed and interpreted the experiment data. H-MY provided the control exome data. C-BX and X-HH interpreted the clinical data. All authors contributed to the final manuscript.

## Conflict of Interest Statement

The authors declare that the research was conducted in the absence of any commercial or financial relationships that could be construed as a potential conflict of interest.

## Footnotes

1 https://www.ebi.ac.uk/arrayexpress/experiments/E-GEOD-30352/

2 http://genome.ucsc.edu/

3 http://bio-bwa.sourceforge.net/

4 http://samtools.sourceforge.net/

5 http://www.ncbi.nlm.nih.gov/snp/

6 http://www.1000genomes.org

7 http://www.ncbi.nlm.nih.gov/refseq/

8 http://www.molgen.ua.ac.be/bioinfo/novosnp/
