# Supplementary material for: Exome Sequencing Identifies TENM4 as a Novel Candidate Gene for Schizophrenia in the SCZD2 Locus at 11q14-21
Source: Front Genet. 2019 Jan 28;9:725. doi: 10.3389/fgene.2018.00725 (PMC6360184; doi:10.3389/fgene.2018.00725)
Supplement: Supplementary file 1 [file Data_Sheet_1.pdf]

Exome sequencing identifies *TENM4* as a novel candidate gene for schizophrenia in the SCZD2 locus at 11q14-21

Chao-Biao Xue[1,2], Zhou-Heng Xu[3], Jun Zhu[1,4], Yu Wu[1], Xi-Hang Zhuang[1], Qu-Liang Chen[1], Cai-Ru Wu[1], Jin-Tao Hu[1], Hou-Shi Zhou[2], Wei-Hang Xie[2], Xin Yi[5], Shan-Shan Yu[5], Zhi-Yu Peng[5], Huan-Ming Yang[5], Xiao-Hong Hong[1]*, Jian-Huan Chen[3]*

1. Mental Health Center, Shantou University Medical College, Shantou, Guangdong, China

2. Shantou Central Hospital, the Affiliated Shantou Hospital of Sun Yat-sen University, Shantou, Guangdong, China

3. Laboratory of Genomic and Precision Medicine, Wuxi School of Medicine, Jiangnan University, Wuxi, Jiangsu, China

4. Shenzhen Kang Ning Hospital, Shenzhen, Guangdong, China

5. Beijing Genomics Institute – Shenzhen, Shenzhen, China

The first two authors contribute to the study equally.

***Correspondence:**

Xiao-Hong Hong,

Mental Health Center, Shantou University Medical College, North Taishan Road, Shantou, Guangdong, China, 515065.

Phone: +86-13802717112, Fax : 86-754-82510525, Email: hongxiaohong@21cn.com.

Jian-Huan Chen,

Laboratory of Genomic and Precision Medicine, Wuxi School of Medicine, Jiangnan University, Wuxi, Jiangsu, China, 214122.

Phone: +86-510-85197101, Email: cjh_bio@hottmail.com.

Supplementary Figure Legend

Figure S1. Sanger sequencing of the *TENM4* mutations in detected in schizophrenia. Chromatography is shown for (A) c.6724C>T detected in the schizophrenia family, and (B) c.5738>G and (C) and c.6880>A which are detected in sporadic schizophrenic patients.

Figure S2. Relative expression level of *TENM4* mRNA in RNA-Seq of 6 tissues from 4 species. RNA-seq of the brain, cerebellum, heart, kidney, liver and testis from human, macaque, mouse and opossum are obtained from the Baseline Atlas of Gene Expression Altas website (http://www.ebi.ac.uk/gxa/experiments/E-GEOD-30352). Relative expression level of *TENM4* mRNA is calculated as the original FPKM (fragments per kilobase of transcript per million mapped reads) normalized by the highest FPKM among the six tissues in each species. In Macaca, the ortholog of human *TENM4* is named *ODZ4*. The data of *FUK* and *SLC11A2* are shown for comparison. Compared to the other two genes, *TENM4* shows highest expression and specificity in the brain across all four species.

Table S1. Summary of original exome sequencing data.

Data	II-1	III-1	III-2*	III-4	Mean
Number of raw reads(M)	102.8	113.6	79.1	113.6	102.3
Average read length(bp)	101	101	101	101	101
Raw data yield(Gb)	10.4	11.5	8.0	11.5	10.3
Number of reads mapped to the genome (M)	76.9	111.5	54.8	111.5	88.7
Fraction of uniquely mapped bases on target(%)	69.5%	74.3%	60.0%	74.3%	69.5%
Data mapped to target region (Gb)	5.4	5.8	3.3	5.8	5.1
Mean depth of target region (fold)	66.6	71.7	40.1	71.7	62.5
Coverage of target region (%)	96.2%	96.3%	95.9%	96.3%	96.2%
Target region with more than 10X (%)	93.1%	93.2%	89.5%	93.2%	92.3%

***The proband**

Table S2. Summary of detected variants in four exomes of the schizophrenia family.

Variants	II-1	III-1	III-2*	III-4
Number of variants (SNVs + indels)	88265+8435	89416+8606	86030+7959	73209 +7561
Synonymous SNVs	10546	10684	10603	10364
Nonsynonymous SNVs	9449	9452	9519	9226
Stopgain SNVs	67	63	76	69
Stoploss SNVs	8	10	8	9
Frameshift indels	91	102	94	97
Nonframeshift indels	236	244	244	186

***The proband**

Table S3. Loci linked to schizophrenia in the online Mendelian inheritance of man database (OMIM).

Linkage locus	OMIM ID	Location	Gene	Genomic coordination
SCZD1	%181510	5q23-q35	unknown	chr5:115,200,000 - 180,915,260
SCZD2	%603342	11q14-q21	unknown	chr11:77,100,000 - 97,200,000
SCZD3	%600511	6p23	unknown	chr6:13,400,000 - 15,200,000
SCZD4	#600850	22q11.21	PRODH	chr22:18,900,205-18,924,065
SCZD5	%603175	6q13-q26	unknown	chr6:70,000,000 - 164,500,000
SCZD6	%603013	8p21	unknown	chr8:19,000,000 - 28,800,000
SCZD7	%603176	13q32	unknown	chr13:95,000,000 - 101,700,000
SCZD8	%603206	18p	unknown	chr18:0 - 17,200,000
SCZD9	#604906	1q42.2	DISC1	chr1:231,762,560-232,177,017
SCZD10	%605419	15q15	unknown	chr15:40,100,000 - 44,800,000
SCZD11	%608078	10q22.3	unknown	chr10:77,700,000 - 82,000,000
SCZD12	%608543	1p36.2	unknown	chr1:7,200,000 - 16,200,000
SCZD13	%613025	15q13	unknown	chr15:28,100,000 - 33,600,000
SCZD14	%612361	2q32.1	unknown	chr2:183,000,000-189,400,000
SCZ15	#606230	22q13.33	SHANK3	chr22:51,113,069-51,171,639
SCZ16	%613959	7q36.3	unknown	chr7:155,100,000-159,138,663
SCZ17	#600565	2p16.3	NRXN1	chr2:50,145,642-51,259,673
SCZ18	#133550	9p24.2	SLC1A1	chr9:4,490,426-4,587,468

Table S4. Gene information for prioritization of variants from filtering pipeline

No.	Symbol	Full name	mRNA Accession Number	Functiona l class of variant	SNV (protein change)	Chromosome location	Adjacent schizophrenia locus	Linked disease in OMIM	Inheritance	Other related functional information
1	*TENM4*	teneurin transmembrane protein 4	NM_001098816	missense	c.6724C>T (p.R2242C)	11q14.1	11q14-22	unknown	NA	NA
2	*SLC11A2*	solute carrier family 11 (proton-coupled divalent metal ion transporter), member 2	NM_001174126	splice-site	c.310-2A>G	12q13	NA	Anemia, hypochromic microcytic	Autosomal recessive	NA
3	*TSPAN12*	Tetraspanin 12	NM_012338	missense	c.32G>A (p.R11H)	7q31.3	NA	Exudative vitreoretinopathy	Autosomal recessive/ Autosomal dominant	NA
4	*FUK*	fucokinase	NM_145059	missense	c.22G>T (p.D8 Y)	7q22.1	NA	unknown	NA	Lack of an apparent phenotype in individuals with loss of function variants*
5	*LTRMM4*	leucine rich repeat transmembrane neuronal 4	NM_194071	missense	c.577A>G (p.K193E)	7q33	NA	unknown	NA	Changed expression level in postmortem schizophrenic patient brain**

* Reference: Alsalem, A. B., Halees, A. S., Anazi, S., Alshamekh, S. & Alkuraya, F. S. Autozygome Sequencing Expands the Horizon of Human Knockout Research and Provides Novel Insights into Human Phenotypic Variation. *PLoS Genet* **9,** e1004030 (2013).

** Reference: An LRRTM4-HSPG Complex Mediates Excitatory Synapse Development on Dentate Gyrus Granule Cells. *Neuron* **79,** 680–695 (2013).

Table S5. Primers used in PCR and Sanger sequencing.

No.	Primer	Exon	Primer Sequence	Amplicon length (bp)	Used in
1	TENM4_R2242CF	32	AGGTAGGACCCTACGCCAAT	234	The schizophrenia family
	TENM4_R2242CR		AGCCATCCTCATCCATCTTG		
2	TENM4_E31F	31	ACTCAAGAGCTTCCCAGCAG	499	Sporadic patients and controls
	TENM4_E31R	31	TGGGTCAGATACAGCCCACT		
3	TENM4_E32-1F	32	TTCCGTCCCTTTAAGTCTCG	664	Sporadic patients and controls
	TENM4_E32-1R	32	CCAAACTTCCCAAACTGCTC		
4	TENM4_E32-2F	32	ACGCCCGTTTTGACTACAAC	692	Sporadic patients and controls
	TENM4_E32-2R	32	GTGGCTGTGGCTGCTCTT		
5	TENM4_E32-3F	32	AGATGGATGAGGATGGCTTC	693	Sporadic patients and controls
	TENM4_E32-3R	32	GAGCACTTTATGGGGTCCTG		
6	TENM4_E33F	33	AGCAGTTGACTCTCCCACCA	543	Sporadic patients and controls
	TENM4_E33R	33	GCAGCCTTGTGCAGGAATTA		

Table S6. Coding variants detected in exomes of the family and 1236 non-schizophrenia controls.

No.	cDNA change	protein change	Exon / total Exons	Consequence type	dbSNP ID	SIFT prediction	PolyPhen prediction	III-1 (A)	III-4 (A)	II-1 (U)	III-2 (U)	MAF in control exomes	Protein region of TENM4
1	c.153C>T	p.D51D	5/34	synonymous	rs61745036	-	-					0.0008	Teneurin N-terminal
2	c.508C>T	p.L170L	7/34	synonymous	novel	-	-					0.0004	Teneurin N-terminal
**3**	**c.973C>T**	**p.R325W**	**9/34**	**missense**	**novel**	**deleterious**	**damaging**					**0.0004**	**Teneurin N-terminal**
4	c.1516G>C	p.E506Q	12/34	missense	rs17137261	deleterious	damaging					0.0012	
5	c.2109C>T	p.H703H	15/34	synonymous	rs73498549	-	-					0.0032	EGF-like
6	c.2286G>A	p.P762P	16/34	synonymous	rs11237621	-	-	+	+	+	+	0.0210	EGF-like
7	c.2381G>A	p.R794K	17/34	missense	rs78279199	tolerated	benign					0.0004	EGF-like
8	c.2421C>T	p.N807N	18/34	synonymous	rs116862565	-	-					0.0008	EGF-like
9	c.2463C>T	p.C821C	18/34	synonymous	rs34863597	-	-					0.0028	EGF-like
10	c.2601G>A	p.P867P	19/34	synonymous	rs12291278	-	-					0.0016	
11	c.2677T>C	p.S893P	19/34	missense	rs188166086	tolerated	benign					0.0004	
12	c.2727A>G	p.I909M	19/34	missense	novel	deleterious	benign					0.0004	
13	c.2789C>T	p.T930I	20/34	missense	rs76283314	deleterious	damaging					0.0004	
14	c.2804C>G	p.P935R	20/34	missense	rs141706152	deleterious	damaging					0.0004	
15	c.2913C>T	p.I971I	21/34	synonymous	rs17826919	-	-			+	+	0.0105	
**16**	**c.3293A>G**	**p.E1098G**	**22/34**	**missense**	**novel**	**deleterious**	**damaging**					**0.0004**	
17	c.3351T>C	p.I1117I	22/34	synonymous	rs200129089	-	-					0.0004	
18	c.3492C>T	p.D1164D	23/34	synonymous	rs689369	-	-					0.0186	
19	c.5238C>T	p.G1746G	29/34	synonymous	rs1792148	-	-					0.0117	YD
20	c.5337G>A	p.A1779A	30/34	synonymous	rs59660398	-	-					0.0061	YD
21	c.5382C>T	p.T1794T	30/34	synonymous	rs79381517	-	-					0.0012	YD
22	c.5535C>T	p.R1845R	31/34	synonymous	rs111604043	-	-					0.0012	YD
23	c.6105C>T	p.D2035D	32/34	synonymous	rs61740650	-	-					0.0004	YD
24	c.6114A>G	p.A2038A	32/34	synonymous	rs61742000	-	-					0.0032	YD
25	c.6627C>A	p.D2209E	32/34	missense	novel	tolerated	benign					0.0004	YD
26	c.6651C>T	p.Y2217Y	32/34	synonymous	rs61747204	-	-					0.0032	YD
**27**	**c.6724G>A**	**p.R2242C**	**32/34**	**missense**	**novel**	**deleterious**	**damaging**	**+**	**+**			**0**	**YD**
28	c.7062T>C	p.D2354D	32/34	synonymous	rs17136977	-	-					0.0012	YD
29	c.7191G>A	p.Q2397Q	32/34	synonymous	rs61745709	-	-					0.0012	
30	c.7509C>T	p.L2503L	33/34	synonymous	rs2277277	-	-			+	+	0.0279	
31	c.7729G>T	p.G2577C	34/34	missense	rs61745704	deleterious	damaging					0.0020	
32	c.7913G>A	p.R2638Q	34/34	missense	rs114100394	tolerated	benign					0.0008	
33	c.7933G>A	p.V2645I	34/34	missense	rs76975254	tolerated	benign					0.0008	
34	c.8000A>G	p.Q2667R	34/34	missense	rs78572681	tolerated	benign					0.0008	
35	c.8011G>A	p.G2671R	34/34	missense	rs114114492	tolerated	benign					0.0004	
36	c.8115C>T	p.R2705R	34/34	synonymous	rs117724179	-	-					0.0004	
37	c.8189G>C	p.S2730T	34/34	missense	novel	tolerated	benign					0.0004	
38	c.8198C>T	p.R2733Q	34/34	missense	rs185503085	tolerated	benign					0.0004	
**39**	c.8203C>T	**p.Q2735X**	**34/34**	**stop-gained**	**novel**	**-**	**-**					**0.0004**	

III-1 and III-4 are affected (A), and II-1 and III-2 are unaffected (U). + denotes heterozygous genotypes. Bold face shows novel variants predicted to be damaging. Protein region annotation is based on information from Uniprot (http://www.uniprot.org)

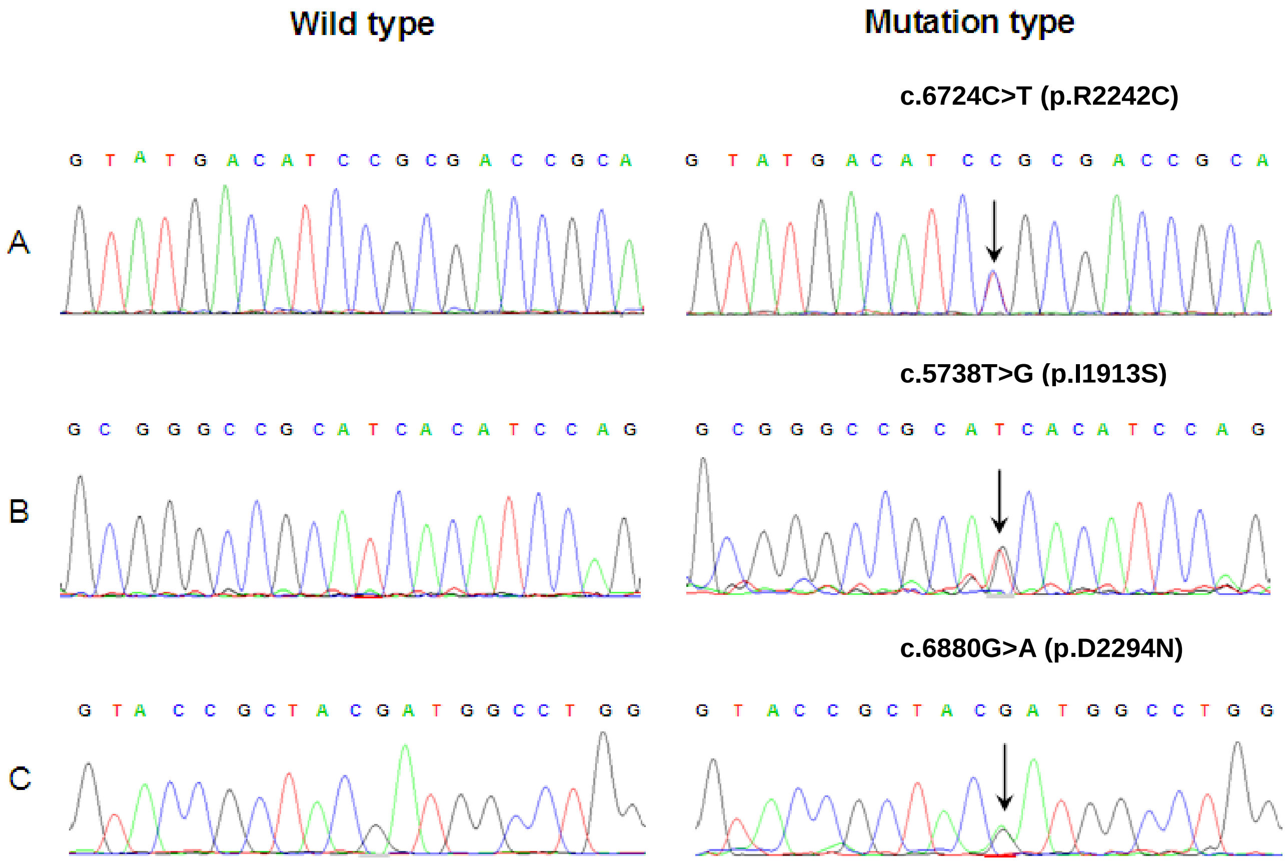

Wild type
Mutation type
c.6724C>T (p.R2242C)
A
G T A T G A C A T C C G C G A C C G C A
G T A T G A C A T C C G C G A C C G C A
c.5738T>G (p.I1913S)
B
G C G G G C C G C A T C A C A T C C A G
G C G G G C C G C A T C A C A T C C A G
c.6880G>A (p.D2294N)
C
G T A C C G C T A C G A T G G C C T G G
G T A C C G C T A C G A T G G C C T G G

Figure S1

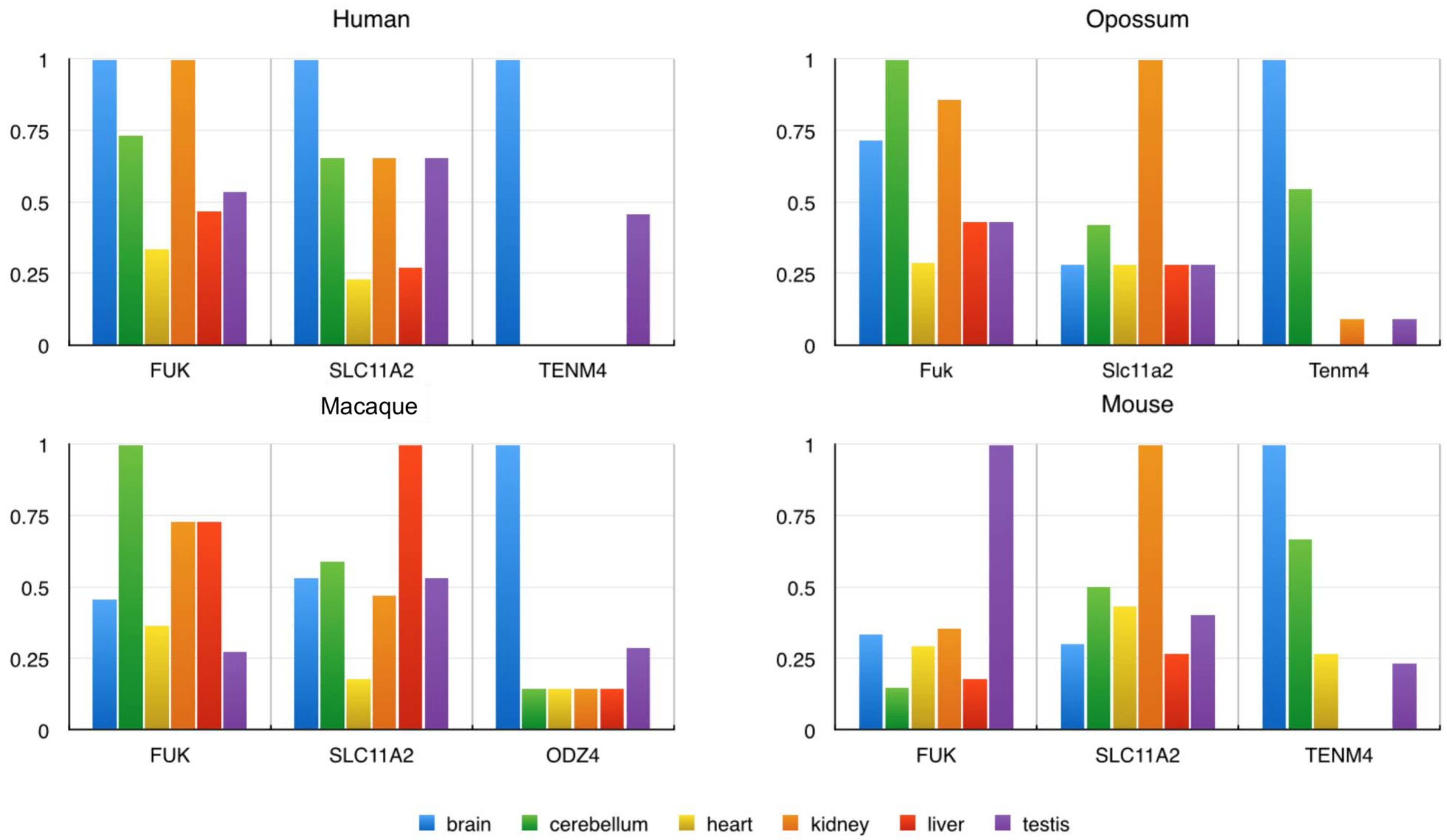
Human
Opossum
Macaque
Mouse
1
0.75
0.5
0.25
0
FUK
SLC11A2
TENM4
Fuk
Slc11a2
Tenm4
ODZ4
brain
cerebellum
heart
kidney
liver
testis

Figure S2
